# Supplementary material for: Master transcription-factor binding sites constitute the core of early replication control elements
Source: EMBO J. 2025 Jul 17;44(16):4499–524. doi: 10.1038/s44318-025-00501-5 (PMC12361434; doi:10.1038/s44318-025-00501-5)
Supplement: Supplementary file 11 — Expanded View Figures [file 44318_2025_501_MOESM11_ESM.pdf]

Expanded View Figures

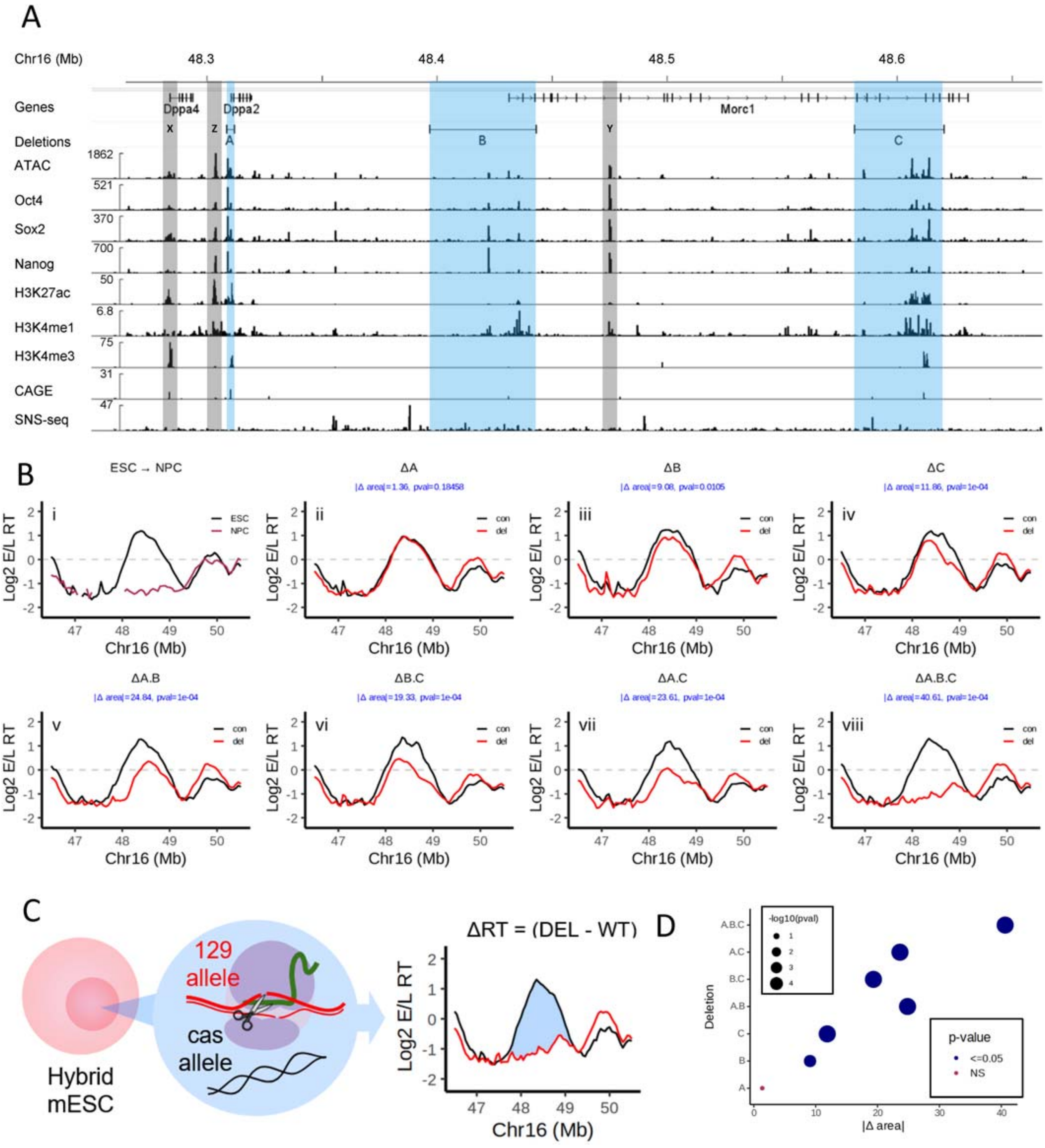

Figure EV1. Epigenetic signature of the Dppa2/4 replication domain ERCEs.

(**A**) IGV browser tracks showing chromatin features of the Dppa2/4 domain (see “Methods” for sources). ERCEs, defined by previous deletions (Sima et al, 2019), are highlighted in blue. X and Y (gray highlights) represent regions that display epigenetic features of ERCEs but did not display ERCE activity in deletion analyses (Sima et al, 2019). (**B**) Log2(E/L) Repli-seq (Marchal et al, 2018) from (Sima et al, 2019). The first panel (i) shows RT before and after mESC (black) to neural precursor cell (NPCs; maroon) differentiation. The remaining panels (ii-viii) show RT profiles from averaged replicates of independent CRISPR clones harboring deletions of one or more ERCEs on one allele (DEL; red) vs. the homologous unmodified allele (CON; black). Individual replicate experiments are shown in Fig. EV3, and the approach to assess the significance of differences in RT between a deletion allele and the corresponding WT allele are shown in Fig. EV2. (**C**) Schematic representation of hybrid mESC model, where one allele contains a deletion and the other serves as control, allowing us to study changes in RT by estimating the area under the ΔRT curve in the DPPA domain (see “Methods”). (**D**) |Δ area| between the averaged RT profile curves of at least two replicates (shown in (**C**)) and the significance of their delay in RT compared to the corresponding WT alleles. Empirical *P* values are shown.

 © The Author(s)

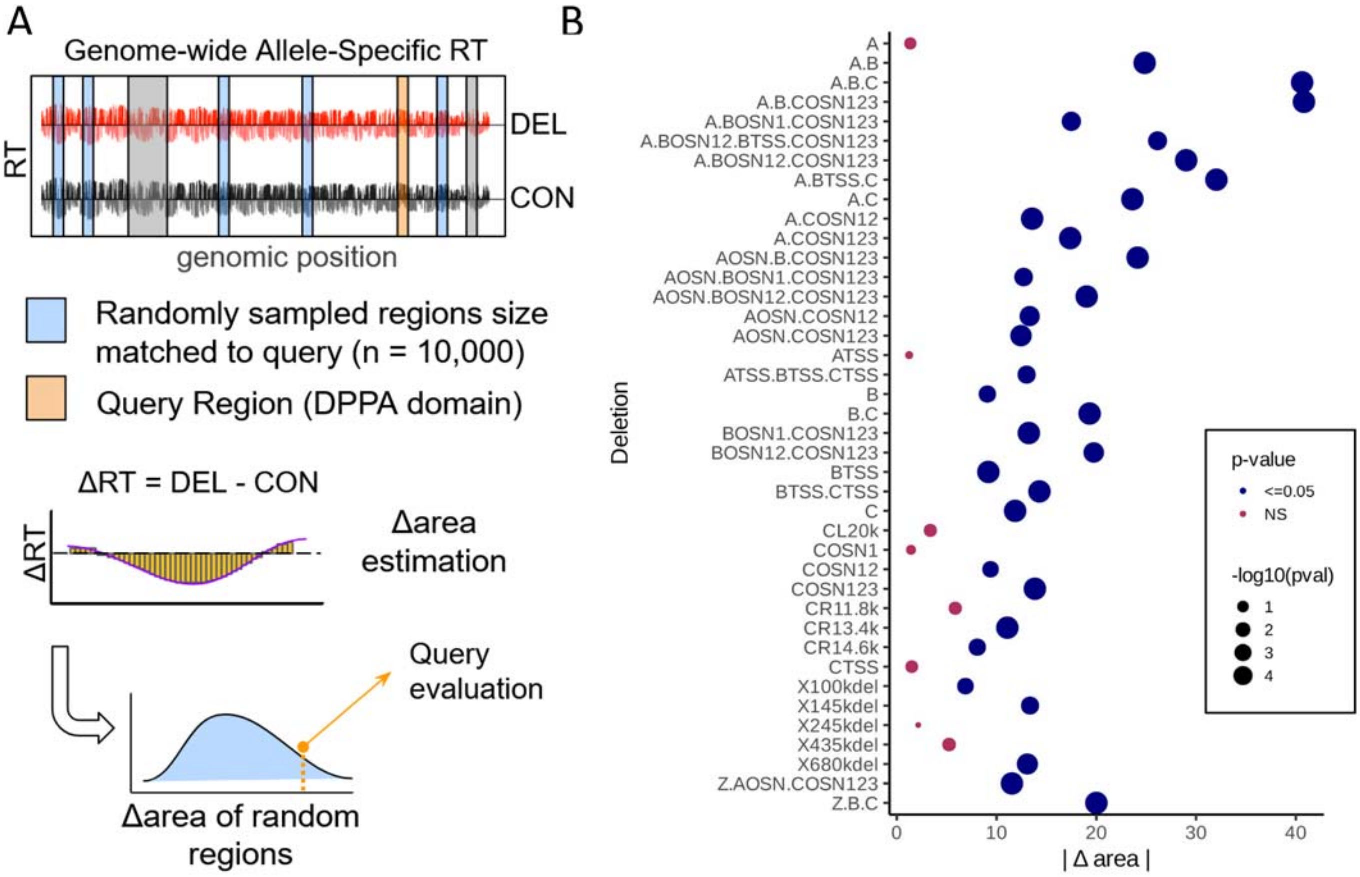

Figure EV2. Statistical analysis of E/L Repli-seq.

(**A**) Schematic representation of AUC approach. (**B**) |Δ area| for all deletions in this study with corresponding *P* values. Color represents significance at *P* value of 0.05 and size represents −log10(*P* value). Empirical *P* values are shown.

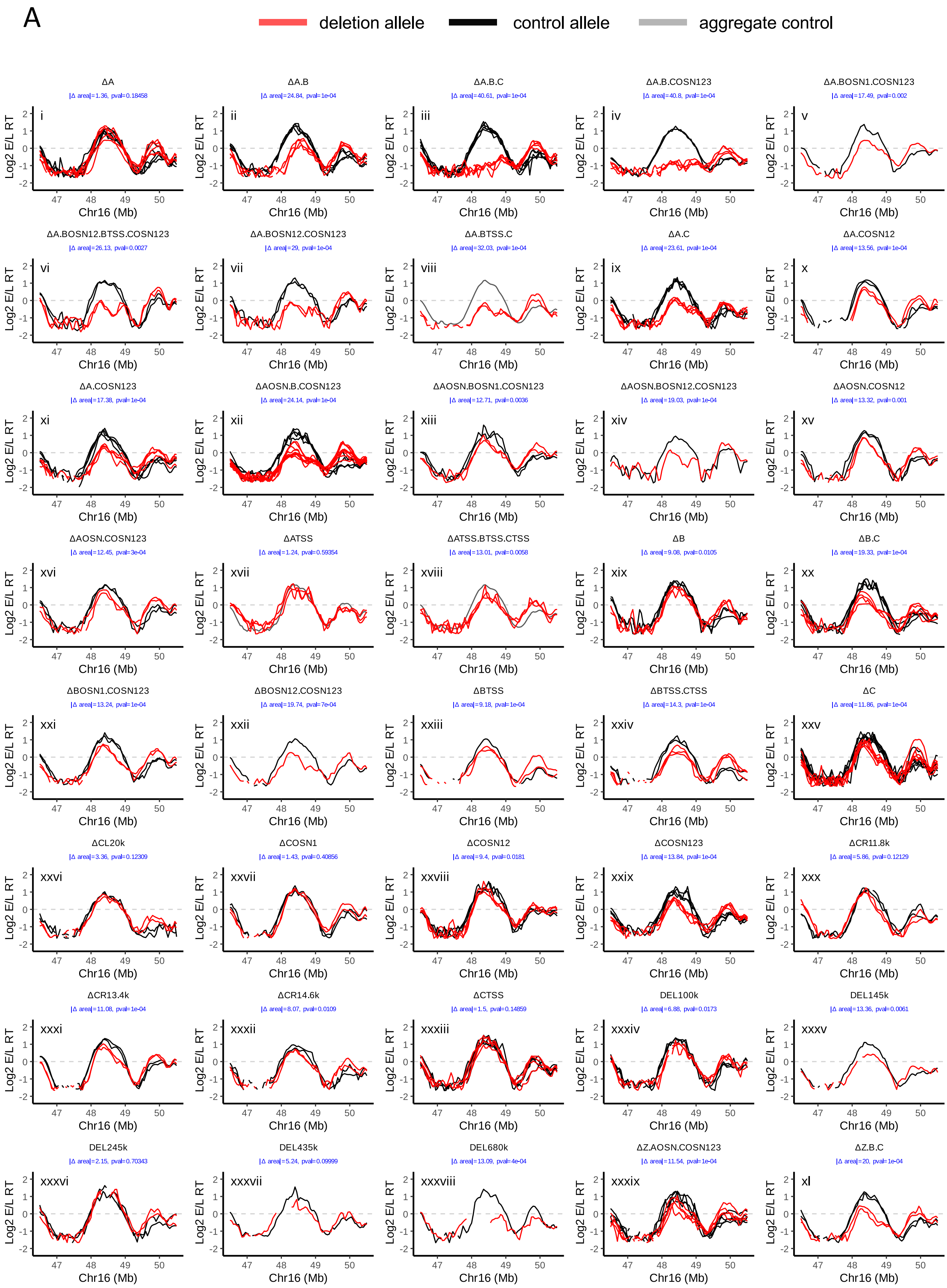
A
deletion allele
control allele
aggregate control
ΔA
|Δ area|=1.36, pval=0.18458
i
ΔA.B
|Δ area|=24.84, pval=1e-04
ii
ΔA.B.C
|Δ area|=40.61, pval=1e-04
iii
ΔA.B.COSN123
|Δ area|=40.8, pval=1e-04
iv
ΔA.BOSN1.COSN123
|Δ area|=17.49, pval=0.002
v
ΔA.BOSN12.BTSS.COSN123
|Δ area|=26.13, pval=0.0027
vi
ΔA.BOSN12.COSN123
|Δ area|=29, pval=1e-04
vii
ΔA.BTSS.C
|Δ area|=32.03, pval=1e-04
viii
ΔA.C
|Δ area|=23.61, pval=1e-04
ix
ΔA.COSN12
|Δ area|=13.56, pval=1e-04
x
ΔA.COSN123
|Δ area|=17.38, pval=1e-04
xi
ΔAOSN.B.COSN123
|Δ area|=24.14, pval=1e-04
xii
ΔAOSN.BOSN1.COSN123
|Δ area|=12.71, pval=0.0036
xiii
ΔAOSN.BOSN12.COSN123
|Δ area|=19.03, pval=1e-04
xiv
ΔAOSN.COSN12
|Δ area|=13.32, pval=0.001
xv
ΔAOSN.COSN123
|Δ area|=12.45, pval=3e-04
xvi
ΔATSS
|Δ area|=1.24, pval=0.59354
xvii
ΔATSS.BTSS.CTSS
|Δ area|=13.01, pval=0.0058
xviii
ΔB
|Δ area|=9.08, pval=0.0105
xix
ΔB.C
|Δ area|=19.33, pval=1e-04
xx
ΔBOSN1.COSN123
|Δ area|=13.24, pval=1e-04
xxi
ΔBOSN12.COSN123
|Δ area|=19.74, pval=7e-04
xxii
ΔBTSS
|Δ area|=9.18, pval=1e-04
xxiii
ΔBTSS.CTSS
|Δ area|=14.3, pval=1e-04
xxiv
ΔC
|Δ area|=11.86, pval=1e-04
xxv
ΔCL20k
|Δ area|=3.36, pval=0.12309
xxvi
ΔCOSN1
|Δ area|=1.43, pval=0.40856
xxvii
ΔCOSN12
|Δ area|=9.4, pval=0.0181
xxviii
ΔCOSN123
|Δ area|=13.84, pval=1e-04
xxix
ΔCR11.8k
|Δ area|=5.86, pval=0.12129
xxx
ΔCR13.4k
|Δ area|=11.08, pval=1e-04
xxxi
ΔCR14.6k
|Δ area|=8.07, pval=0.0109
xxxii
ΔCTSS
|Δ area|=1.5, pval=0.14859
xxxiii
DEL100k
|Δ area|=6.88, pval=0.0173
xxxiv
DEL145k
|Δ area|=13.36, pval=0.0061
xxxv
DEL245k
|Δ area|=2.15, pval=0.70343
xxxvi
DEL435k
|Δ area|=5.24, pval=0.09999
xxxvii
DEL680k
|Δ area|=13.09, pval=4e-04
xxxviii
ΔZ.AOSN.COSN123
|Δ area|=11.54, pval=1e-04
xxxix
ΔZ.B.C
|Δ area|=20, pval=1e-04
xl
Log2 E/L RT
Chr16 (Mb)

 © The Author(s)

Q16

Figure EV3. All Repli-seq datasets analyzed in this study.

(A) (i–xl) Allele-specific E/L Repli-seq for all the data generated and analyzed in this study, including relevant deletions from our previous publication (Sima et al, 2019). Replicates (independent CRISPR-mediated deletion clones) are shown as different lines in each plot, comparing the deletion (red) allele to the homologous unmodified allele (black). In the occasional cases where a clone suffered one of the multiple deletions in both alleles we use an aggregated WT control (gray). The aggregated control was generated by averaging all RT profiles from the WT alleles across different deletions. The replication domain to the right of Dppa (c16: 49.65–50.05 Mb mm10) shows some clonal variation in RT but there is a poor correlation ($R = -0.35$) between the effects of the deletions in the Dppa domain to this variation. Rather, this domain has been shown to display a genetic difference in RT between *m. castaneus* and *m. musculus* (Rivera-Mulia et al, 2018) as well as high cell to cell variability in RT (Zhao et al, 2020).

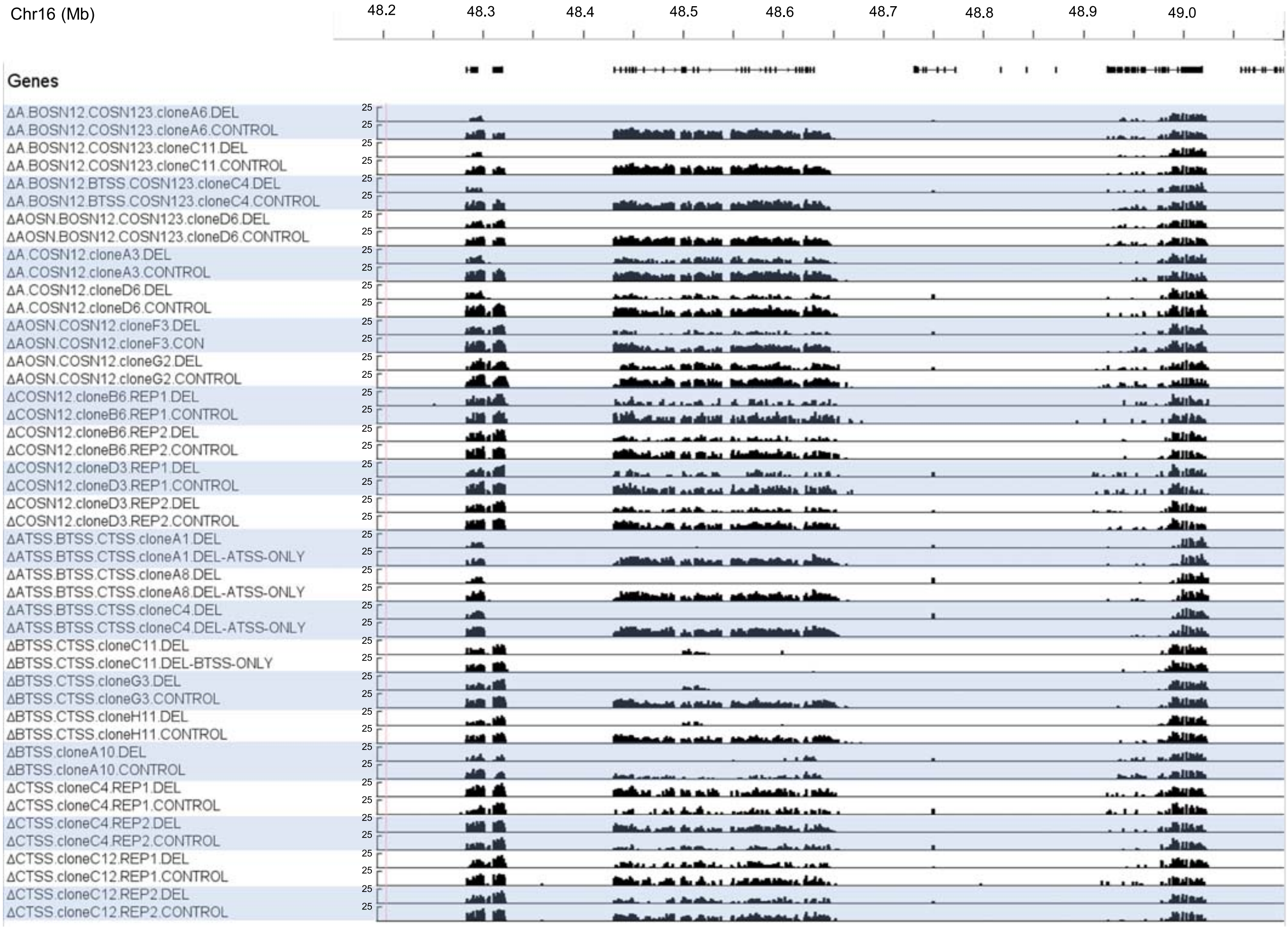

Figure EV4. All Bru-seq datasets generated in this study.

All of the BrU-seq generated in this study, displayed as in Fig. 4. OSN deletion series data tracks are found on the top half while TSS deletion series data tracks can be found on the bottom half. We observed some variation in gene expression between the two alleles in WT cells (Fig. 4). This variation can also be seen in the CTSS deletions (bottom tracks), as clone (C4) harbors a deletion of the *castaneus* allele whereas the other clone (C12) harbors a deletion on the *musculus* allele, and neither CTSS deletion has an effect on Morc1 expression.

 © The Author(s)

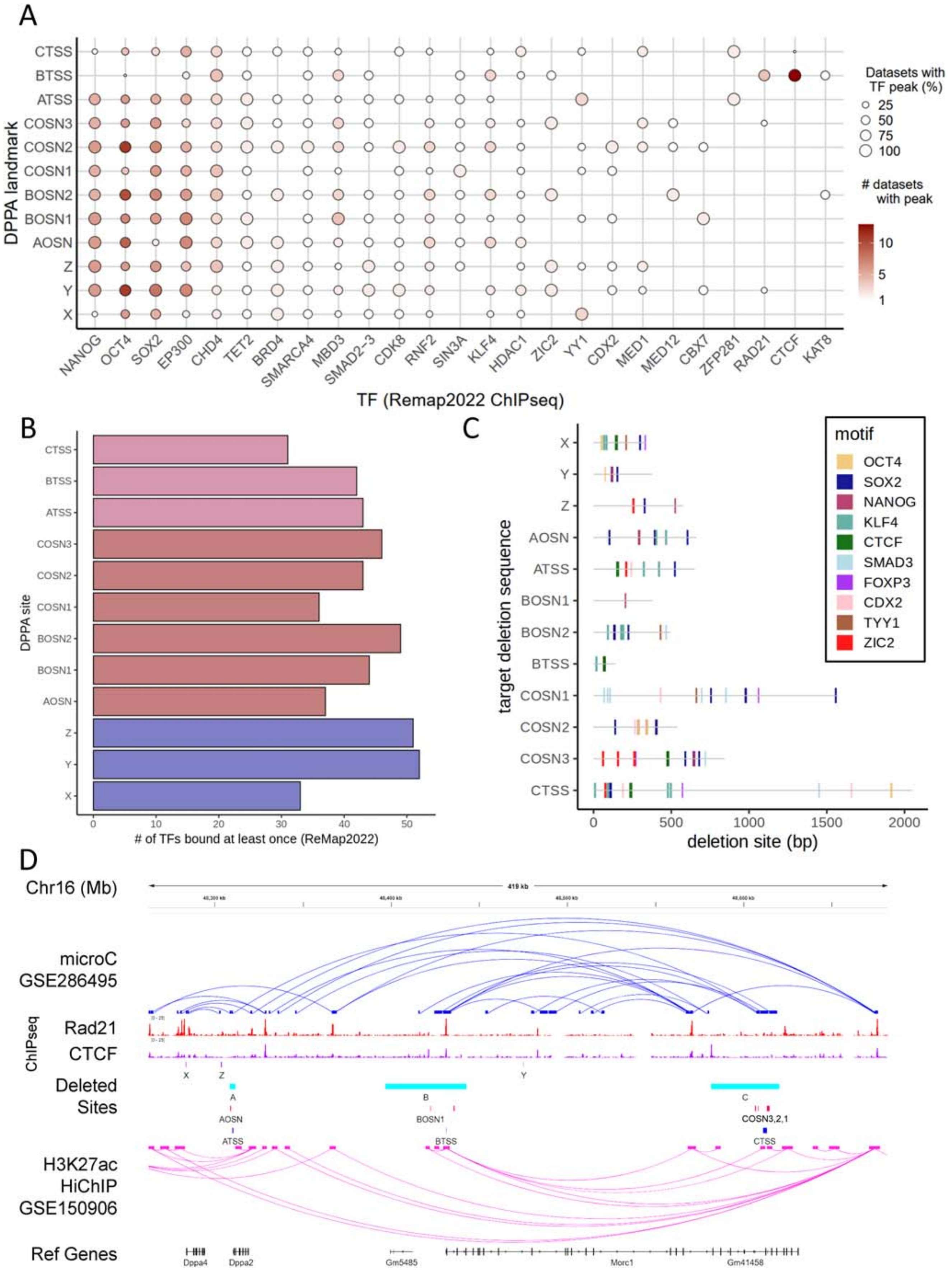
A
DPPA landmark
CTSS
BTSS
ATSS
COSN3
COSN2
COSN1
BOSN2
BOSN1
AOSN
Z
Y
X
NANOG OCT4 SOX2 EP300 CHD4 TET2 BRD4 SMARCA4 MBD3 SMAD2-3 CDK8 RNF2 SIN3A KLF4 HDAC1 ZIC2 YY1 CDX2 MED1 MED12 CBX7 ZFP281 RAD21 CTCF KAT8
TF (Remap2022 ChIPseq)
Datasets with TF peak (%)
25
50
75
100
datasets with peak
10
5
1
B
DPPA site
# of TFs bound at least once (ReMap2022)
0 10 20 30 40 50
C
target deletion sequence
deletion site (bp)
0 500 1000 1500 2000
motif
OCT4
SOX2
NANOG
KLF4
CTCF
SMAD3
FOXP3
CDX2
TYY1
ZIC2
D
Chr16 (Mb)
419 kb
microC GSE286495
ChIPseq
Rad21
CTCF
Deleted Sites
X
Z
Y
A
B
C
AOSN
BOSN1
COSN3,2,1
ATSS
BTSS
CTSS
H3K27ac HiChIP GSE150906
Ref Genes
Dppa4
Dppa2
Gm5485
Morc1
Gm41458

◀ **Figure EV5. Deleted sites are diverse TFBS.**

(**A**) ChIP-seq peaks from 'mESC' datasets in Remap2022 overlapping at least two of the sites of interest in the DPPA domain. The size of the dots indicates the percentage of available ChIP datasets with a peak directly overlapping the site, and the color indicates the number of datasets with peak. (**B**) TF-binding diversity (Hammal et al, 2022) quantified as the number of TFs with at least one ChIP-seq peak from Remap2022 'mESC' overlapping the sites of interest. (**C**) Putative TFBS in target deletion sequences determined by motif presence. Due to the presence of over 400 TF-binding motifs in this collection of elements, only motifs for TFs from the ChIP-seq targets shown on (**A**) are included. (**D**) Micro-C (preprint: Jusuf et al, 2025) and H3K27ac HiChIP loops (Kraft et al, 2022) in the Dppa domain, as well as CTCF and Rad21 ChIP-seq signal over input (Cattoglio et al, 2019; Hansen et al, 2017). Loop resolution varies between datasets and is indicated on *y* axis.

 © The Author(s)
